# Viral particles of the endogenous retrovirus ZAM from *Drosophila melanogaster *use a pre-existing endosome/exosome pathway for transfer to the oocyte

**DOI:** 10.1186/1742-4690-3-25

**Published:** 2006-05-09

**Authors:** E Brasset, AR Taddei, F Arnaud, B Faye, AM Fausto, M Mazzini, F Giorgi, C Vaury

**Affiliations:** 1 INSERM, U384, Faculté de Médecine, BP38, 63001 Clermont-Ferrand, France; 2 Centre of Electron Microscopy, Department of Environmental Sciences, Tuscia, University Viterbo, Italy

## Abstract

**Background:**

Retroviruses have evolved various mechanisms to optimize their transfer to new target cells via late endosomes. Here, we analyzed the transfer of ZAM, a retroelement from *Drosophila melanogaster*, from ovarian follicle cells to the oocyte at stage 9–10 of oogenesis, when an active yolk transfer is occurring between these two cell types.

**Results:**

Combining genetic and microscopic approaches, we show that a functional secretory apparatus is required to tether ZAM to endosomal vesicles and to direct its transport to the apical side of follicle cells. There, ZAM egress requires an intact follicular epithelium communicating with the oocyte. When gap junctions are inhibited or yolk receptors mutated, ZAM particles fail to sort out the follicle cells.

**Conclusion:**

Overall, our results indicate that retrotransposons do not exclusively perform intracellular replication cycles but may usurp exosomal/endosomal traffic to be routed from one cell to another.

## Background

A small group of LTR-retrotransposons from insects is very similar in structure and replication cycle to mammalian retroviruses [1]. They contain three open reading frames, the first two of which correspond to retroviral *gag *and *pol *genes, whereas the third one, ORF3, is a retroviral *env *gene whose function is still unknown. ZAM is one of these retroviruses present in *Drosophila melanogaster *[2]. Its replication cycle is generally absent in flies but a line called "U" exists in which it is highly expressed and gives rise to multiple ZAM proviral copies inserting the germ line. A mutation located on the X-chromosome (X^U^) of the "U" line is responsible for this active expression of ZAM while the wild type X-chromosome (X^S^) is not [3]. ZAM particles from "U" ovaries assemble in a somatic cell lineage of the posterior follicular epithelium and gain access to the oocyte to affect the maternal germ line [4]. These data indicate that ZAM viral particles are capable of exiting the cell where they are assembled and subsequently enter a recipient surrounding cell. Since the mechanisms mediating this viral cell transfer are still unknown, it is uncertain whether viral *env *products could potentially fulfil this role. No enveloped viruses have so far been detected by electron microscopy (TEM) neither as budding particles from the follicle cells nor in the perivitelline space surrounding the oocyte. However, a closely related transposon of *Drosophila melanogaster, gypsy*, has been shown to be transferred from cell-to-cell in the absence of any *env *products [5].

Amongst the mechanism(s) controlling retroviral release from the plasma membrane, the possibility that certain retroviruses could bud intracellularly should also be considered. It is known that HIV and other retroviruses can undergo internal budding by conveying viral particles to multivesicular bodies (MVBs) [6,7]. Virions that bud intracellularly can apparently be released from cells when the endosomal compartments fuse with the plasma membrane [8,9]. Interestingly, previous studies on the ZAM replication cycle provided evidence that vesicular traffic and yolk granules could play such a role in transferring ZAM viral particles to the oocyte [4]. Indeed, ZAM particles were seen to accumulate along the apical border of the ovarian follicle cells in association with yolk polypeptide and vitelline membrane precursors. This observation suggested that ZAM could benefit of this intracellular traffic to get out of the follicle cells during secretion of the vitelline membrane [4].

In this paper, we analyze the mechanism(s) by which ZAM particles are transferred to the oocyte and verify whether this may depend on the process of vitelline membrane secretion and vitellogenin uptake. ZAM particles of a U-line were studied in genetic backgrounds mutated for genes involved either in exosomal traffic of vitelline membrane precursors from the follicle cells, or in the endosomal traffic controlling vitellogenin entrance into the oocyte. By confocal and electron microscope analyses, we show that this exocytosis/endocytosis pathway provides an efficient mechanism for directing ZAM transport from the follicle cells to the oocyte.

## Results

**Figure 1:**
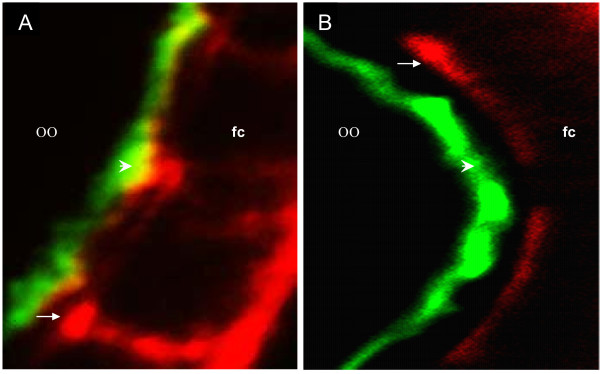
The Gag product of ZAM is restricted to the follicle cells when communication between the follicle cell and the oocyte is blocked. Double staining with Gag anti-body of ZAM (red) and YL1 antibodies (green) of stage 10 ovarian chambers. A) In ovaries of a U-line, the Gag protein of ZAM is detected in follicle cells (red staining), and colocalized with the yolk protein receptor (green) at the oocyte border (yellow). B) In [X^u^/X^u^; fs(2)A17/fs(2)A17] ovaries, the Gag product is restricted to the follicle cells. oo, oocyte; fc, follicle cells.

To elucidate the mechanism involved in ZAM transport, the *fs(2)A17 *mutation was tested in a first set of experiments [10]. Ovarian chambers from *Drosophila *females homozygous for *fs(2)A17 *develop normally until yolk deposition commences, but start to degenerate afterwards [11]. While the oocyte remains in a previtellogenic condition, the columnar follicle cells continue to differentiate, forming abnormal gap junctional contacts with the oocyte. ZAM viral particles are expressed by a cluster of these columnar follicle cells positioned along the posteriormost end of stage 9–10 ovarian chambers, released into the perivitelline space and eventually allowed to enter the oocyte [4]. Thus, ovaries dissected from females with the genotype [X^u^/X^u^; fs(2)A17/fs(2)A17] were examined by confocal microscopy to verify whether this mutation might alter the transport of ZAM particles to the oocyte. Ovaries were double-stained with antibodies against the Gag protein of ZAM and the yolk protein receptor. As expected, Gag proteins in wild type females [X^u^/X^u^; +/+] can be detected at the posterior end of stage 10 follicles, along the follicle cell-oocyte border. Co-localization of Gag with the yolk protein receptor at this cell site is consistent with the hypothesis that Gag-containing particles may indeed be moving from one cell type to another across the perivitelline space. By contrast, Gag remains restricted to the follicle cells and no amount can be detected along the follicle cell-oocyte border in females expressing the mutated genotype [X^u^/X^u^; fs(2)A17/fs(2)A17] (Fig. 1).

**Figure 2:**
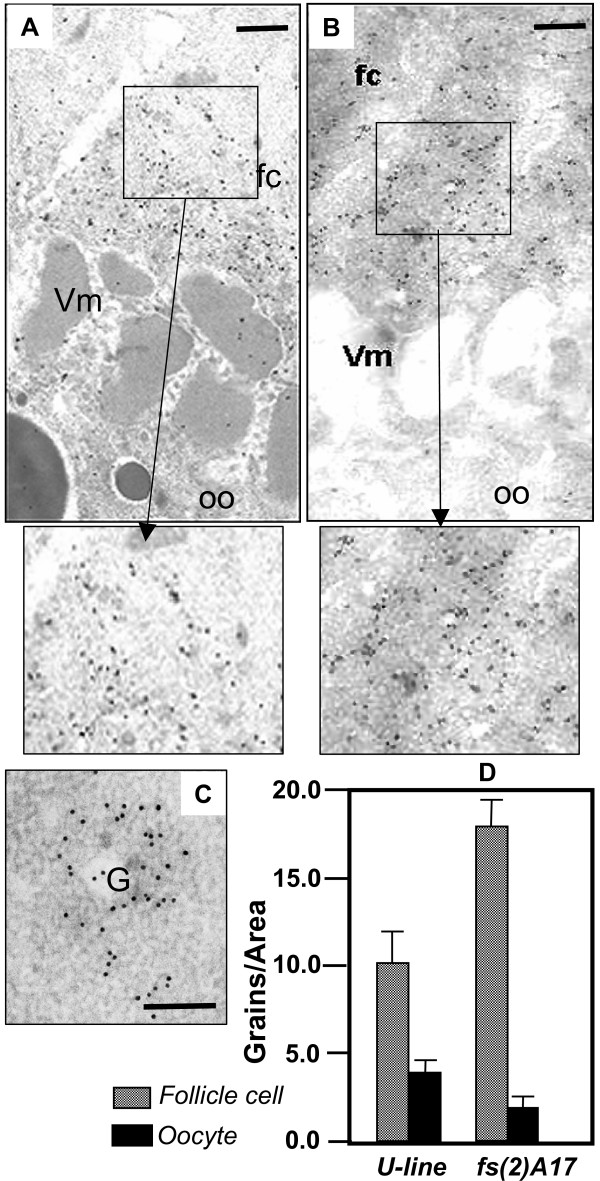
Viral particles of ZAM are restricted to the apical end of the follicle cells in a homozygous fs(2)A17 environment. **A, **The follicle cell/oocyte interface of a U-line stage 9 ovarian follicle is reminded [4]: Viral particles revealed by anti-gag antibodies are detected along the apical end of the follicle cell cytoplasm, on the vitelline membrane and, to a minor extent, in the cortical oocyte. Yolk granules are clearly detected as dark grey circles within the ooplasm. An enlargement of the area defined by the black rectangle is presented below Fig. A. **B, **In a homozygous mutant fs(2)A17, viral particles accumulate in the follicular epithelium, while the vitelline membrane and the oocyte have no viral particles. No yolk granules are visualized within the ooplasm of this mutant line (Scale Bar, 330 nm). An enlargement of the area defined by the black rectangle is presented below Fig. B. **C, **A region of the follicle cell cytoplasm containing the Golgi apparatus as tested with anti-Gag antibodies (Scale Bar, 100 nm). **D**, Histogram expressing the distribution of gold anti-gag tagged grains detected in a U-line bearing or not the fs(2)A17 mutation. Gold grains were counted in the follicle cells and the oocyte comprised within a 0.8 × 1.6 μm reptangular frame bridging the perivitelline space. Data were elaborated using an image analyzer. Standard deviations are reported as bars. fc: follicle cell; G: Golgi apparatus; oo: oocyte; Vm: vitelline membrane.

Since confocal images do not allow to precisely localize ZAM particles at the apical end of the follicle cells, and the posterior pole of the oocyte, we undertook a more discriminative approach through electronic microscopy (EM). Female ovaries mutated or not for *fs(2)A17 *were examined (Fig. 2A and 2B). The presence of ZAM viral particles in different ovarian districts could be easily revealed by immunocytochemistry with gold tagged anti-Gag antibodies. When follicle cells females of the U-line were exposed post-embedding to anti-gag antibodies, gold particles appeared preferentially associated with the apical end of the follicle cell cytoplasm and partly overlapped with the vitelline membrane along the perivitelline space ([4], and Fig. 2A). Gold particles could also be detected in the cortical cytoplasm, especially along the oolemma and on the forming yolk granules. As opposed to these females, and in line with the results of the confocal analysis, a heavy accumulation of ZAM viral particles was visualized only along the apical follicle cytoplasm (Fig. 2B). Very few particles could be detected in the cortical ooplasm of females mutated for *fs(2)A17*, and rare gold grains could occasionally be detected along the forming vitelline envelope (Fig. 2B). In the follicle cytoplasm, ZAM viral particles appeared to be associated with secretory granules as well as accumulated at the apical pole of follicle cells as revealed by the accumulation of gold grains in both these follicle cell regions (Figs. 2B and 2C]. Viral particle distribution in these ovaries was quantified by determining the extent of anti-gag labelling across the follicle cell/oocyte interface. The histogram depicted in Fig. 2D clearly shows that follicle cell labelling is highly enhanced in *fs(2)A17 *ovaries whereas ooplasm labelling is decreased. These observations are in line with the expected phenotypes of the mutant whereby viral particles accumulate in the follicular epithelium when vitellogenic development is arrested as in *fs(2)A17 *flies.

Based on these observations it may be concluded that occurrence of abnormal junctional coupling along the follicle cell/oocyte interface greatly interferes with the release of ZAM viral particles from the follicle cells.

Transfer of ZAM particles was subsequently examined in flies unable to secrete yolk proteins (YPs) from the ovarian follicle cells and fat body cells [19]. Females homozygous for the *fs(1)1163 *mutation are sterile at 18°C, while females heterozygous are sterile at 29°C [12]. In both cases, females produce flaccid eggs which never develop, due to failure of the yolk polypeptide YP1 to be secreted from the ovarian follicle cells and fat body cells [13]. So even though the remaining yolk proteins (YPs) are secreted from both tissues, they precipitate in the intercellular spaces of the follicular epithelium, giving rise to such abnormal structures as globules and crystalline fibers [14].

**Figure 3:**
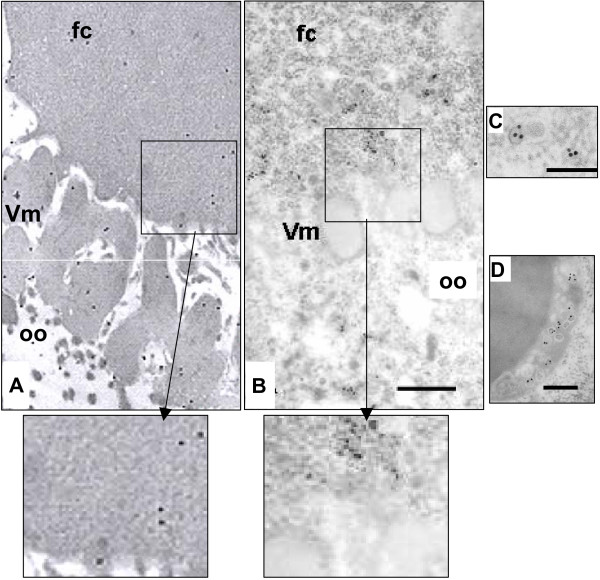
When the yolk protein 1 (YP1) is mutated, ZAM particles are frequently visualized in association with the Golgi apparatus in the follicle cells, and in the superficial layer of the yolk granules in the oocyte. **A and B, **stage 9 ovarian chambers heterozygous X^U^/X^S ^and X^U^/*fs(1)1163 *respectively, show fewer than normal anti-Gag binding sites in the follicle cells than X^U^/X^U ^ovarian chambers (see Fig 1A) (Scale Bar, 400 nm). An enlargement of the area defined by the black rectangle is presented below Fig. A and B. ZAM viral particles are preferentially associated with the Golgi apparatus in the follicle cells as presented in **C **(Scale Bar, 100 nm), or with the yolk granules in the cortical ooplasm as presented in **D **(Scale Bar, 100 nm). Legend is as in figure 2.

Since the *f(1)1163 *mutation and the genetic determinant activating ZAM expression are both located on the X-chromosome, heterozygous females were generated with the [X^S^/X^U^] genotype. Ovaries dissected from X^S^/X^U ^females wild type or mutated for *fs(1)1163 *(Fig 3A and 3B respectively] exhibit fewer than normal ZAM viral particles along the follicle cell/oocyte border (compare Fig. 3A and 3B to Fig. 2A). This can be easily explained by the heterozygous status of the X^U ^chromosome in these females as already reported by Desset *et al*. [3]. Nevertheless, as revealed by anti-Gag immunostaining, ZAM viral particles did not preferentially accumulate at the apical end of the follicle cells of the *fs(1)1163 *mutant line but rather were detected intra-cytoplasmically most frequently included in regions of the Golgi apparatus (Fig. 3C). Inside the oocyte, ZAM viral particles were only rarely seen in the cortical ooplasm, occurring preferentially in association with the yolk granules (Fig. 3D). Thus, a default in YP secretory products is correlated with a default in ZAM particles localization at the apical side of the plasma membrane of follicle cells.

Finally, we asked whether transfer of ZAM particles to the oocyte could be prevented in case endocytosis is impaired by lack of a specific yolk protein receptor. Earlier ultrastructural analyses of *Drosophila *female mutants for *yolkless *(*yl*) had clearly shown that vitellogenic oocytes require expression of the *yl *gene to sustain endocytic activity [15,16]. Female flies homozygous for this gene or heterozygous for the strong allele *yl*^- ^produce oocytes with much less than normal coated pits and vesicles in the cortical ooplasm. Molecular characterization of the *yl *gene has demonstrated that this mutated phenotype can be attributed to lack or reduced expression of the yolk protein receptor along the oocyte plasma membrane [25].

**Figure 4:**
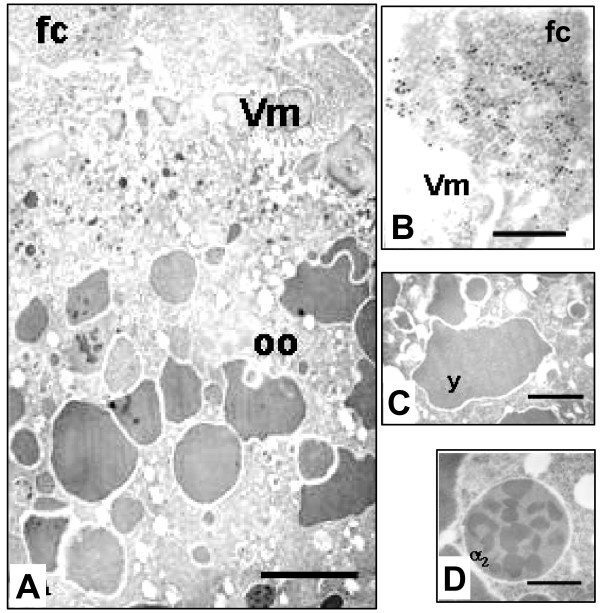
ZAM particles accumulate along the apical end of the follicle cells when the yolk protein receptor *yl *is mutated. **A, **a stage 9 ovarian chamber from a *Drosophila *female fly heterozygous for *yolkless *sectioned along the posterior pole to show those columnar follicle cells that are expected to express ZAM viral particles (Scale Bar, 1 μm). **B**, numerous presumptive ZAM viral particles, some of which are heavily gold-labeled following exposure to anti-gag antibodies, are visible along the apical end of a follicle cell and in close association with secretory granules containing the vitelline membrane precursors (Scale Bar, 200 nm). **C**, an abnormally shaped yolk granule in the cortical ooplasm of *yl *oocytes. Note that this granule has neither a superficial layer nor any ZAM viral particles associated (Scale Bar, 500 nm). **D**, an alpha 2 yolk granule from a stage 10 ovarian chamber of a yl- fly (scale bar, 500 nm). Fc; follicle cells; Vm: vitelline membrane; y: yolk granules; α2: alpha 2 yolk granule.

When stage 9–10 ovarian chambers were allowed to express ZAM in a heterozygous [X^u^/yl^-^] genotype, no viral particles were ever detected in the cortical ooplasm, neither along the oocyte plasma membrane nor in association with the yolk granules (Fig. 4A and 4C). As expected, yolk granules in *yl*^- ^oocytes were abnormally shaped, having no superficial layer along the entire periphery, nor any ZAM viral particle associated with it (Fig. 4A and 4C). That vitellogenesis was somehow abnormal in these mutant oocytes could also be deduced from the early appearance of alpha 2 yolk spheres in stage 10 ovarian chambers, rather than from stage 12 onwards as it should occur in wild type ovaries (Fig. 4D). Regardless of the ultimate size and shape attained by the yolk granules in *yl*^- ^oocytes, none of them was ever found associated with ZAM viral particles. In the follicle cells, gold tagged grains were preferentially seen in association with secretory granules (Fig. 4B). These data indicate that impairment of the endocytic traffic in oocytes of heterozygous *yolkless *mutants prevents ZAM viral particles from acceding into the cortical ooplasm. Since ZAM particles egress from the follicle cells is greatly impaired, a causal relationship is likely to exist in *Drosophila *between the pathway joining the follicular epithelium with the oocyte and the endocytic uptake of vitellogenin.

## Discussion

Retroviruses have evolved a variety of different mechanisms to optimize their transfer into new target cells through late endosomes [17]. Here we show that a connection exists between the traffic of ZAM viral particles and the endosomal trafficking of vitellogenin from the follicle cells to the oocyte in *Drosophila *oogenesis.

1- The YP1 protein is required for targeting ZAM particles to the apical end of the follicle cells:

In the first step of infection, the viral genomic material is directed toward the apical plasma membrane where particles are released from the cell. The use of mutations affecting synthesis of yolk protein 1 (YP1) has shown that a fully functional secretory apparatus is required for ZAM particles to be targeted along the apical border of the follicle cells. In fact, when YP1 is mutated, as in *fs(1)1163 *females, ZAM particles are frequently visualized intra-cytoplasmically in association with the Golgi apparatus. This could either be a direct consequence of the reduced secretory activity of the follicle cells or, alternatively, it could be the absence of YP1 itself that impedes ZAM viral particles to reach the apical end of the follicle cells. In any case, secretory granules and their associated yolk proteins are important factors in controlling the release of viral particles from the Golgi apparatus and targeting them toward the apical pole of the follicle cells. A parallel can be made between these data and a study performed on a mammalian retrovirus: the murine leukaemia virus (MLV) [2]. Indeed, Basyuk et al. (2003) have shown that MLV viral prebudding complexes containing Env, Gag and retroviral RNAs are formed on endosomes, and subsequently routed to the plasma membrane. Thus, ZAM particles transport via the YP secretory products brings another example in which tethering to vesicles help for directing RNA transport.

Expression of the mutant *fs(1)1163 *allele does affect not only YP1 secretion, but also the rate at which YPs are processed during vitellogenesis within the ooplasm [12]. In our experiments, this down-regulation or even arrest of vitellin processing in the yolk granules [14] has been found associated with a higher accumulation of ZAM viral particles in the superficial layer of the yolk granules in the oocyte. However, from our current data it is unknown whether ZAM viral particles may accumulate as unprocessed products in the yolk superficial layer or simply be re-distributed in fewer than normal yolk granules.

2 – The transfer of ZAM particles requires a close contact between the plasma membranes of the follicle cells and the oocyte:

The second step in the traffic of viral particles is to sort out of the cells where they assemble. This transfer of ZAM particles occurs when the oocyte is undergoing endocytosis for vitellogenin uptake. In insect wild type ovaries, junctional communications between the follicle cells and the oocyte are required for germ cell differentiation [18] and vitellogenin uptake into nascent yolk spheres [19]. This relationship has actually been proved in *Oncopeltus fasciatus *by Anderson and Woodruff (2001) who found that a junctionally diffusible molecular signal has to be transferred from the follicle cells to the oocyte for vitellogenin to be taken up endocytically and conveyed to the yolk granules. Our data show that release and transfer of ZAM particles from the follicle cells to the oocyte are blocked in *fs(2)A17 *flies with abnormally shaped gap junctional contacts, thus indicating that establishment of proper interactions at this cell juncture is a precondition for ZAM viral particles to gain access to the oocyte. It has recently been reported that retroviruses are preferentially released along membrane sites where cell-to-cell contacts occur [20-22]. These sites of cell/cell contacts, also termed virological or infectious synapses, express high concentrations of adhesion molecules (Integrins, LFA) and talin, which are known to link adhesion rings to the actin cytoskeleton, as well as to cause polarization of the microtubule organization center (MTOC) toward the synapse itself [23]. Since cell-cell communication along the follicle cells/oocyte border is also required for efficient ZAM transfer to the oocyte, it can be hypothesized that open gap junction channels between the follicular epithelium and the oocyte are required to render "infectious synapses" active for the transfer of ZAM particles. Interestingly, such a direct cell-cell transfer would localize ZAM particles to the MTOC, allowing particles to exploit the microtubule network and be transferred from the posterior pole of the oocyte to the anterior one close to the germ cell nucleus.

Alternatively, an earlier research performed on ZAM replication cycle had led to the detection of ZAM particles within the secretory granules of the follicle cells [16]. If cell-cell communication along the follicle cells/oocyte is disrupted due to mutated gap junctions [24], exocytosis of vitellogenin granules is then impaired and their associated ZAM particles cannot escape from the follicle cells. Although both scenarios are not mutually exclusive, the latter view could explain more explicitly why ZAM particles can be found in the intercellular space between the follicle cells and the oocyte.

3 – Impairment of the endocytic traffic in the oocyte disturbs ZAM viral particles transit to the oocyte.

When released extracellularly, ZAM viral particles will ultimately enter the oocyte. We have shown that impairment of the endocytic traffic in the oocyte due to a mutation affecting the yolk protein receptor *yolkless *prevents ZAM viral particles from acceding into the cortical ooplasm. There are at least three well-described mechanisms for internalizing proteins from the plasma membrane, including endocytosis via clathrin-coated pits, caveolae, and rafts. A close examination of wild type oocytes has clearly shown that anti-Gag binding sites in the cortical ooplasm coincide neither with the coated pits nor with the coated vesicles [16], indicating that ZAM viral particles are likely to enter the oocyte by alternative pathways, perhaps by using the pathway provided by caveoles. Interestingly, a number of recent reports have clearly demonstrated that both the simian virus 40 virus [25] and the HIV [26] can be actually internalized into competent cells by caveolar endocytosis. This is also consistent with the role currently attributed to the caveolae as plasma membrane microdomains functionally distinguishable from endocytotic trafficking [27]. In fact, in our previous finding ZAM viral particles could never be detected in association with peroxidase-labelled endocytic vesicles [4]. The absence of viral particles in the oocyte should not necessarily imply any factual impediment for the virus entry. Viral particles could still be entering the oocyte, but remain undetected due to the yolk granule incapability to store and process them. Yolk granules in *yolkless *ovaries are in fact abnormally shaped and void of any structural component in the superficial layer, a condition that could lead to an uncontrolled yolk polypeptide processing. It should be recalled here that yolk granules of insect oocytes are functionally equivalent to multivesicular bodies, a cell organelle that in infected cells may serve as an intracellular compartment to process viral complexes and direct them to other cell sites, including the plasma membrane [28].

## Conclusion

Overall, our study shows that transfer of ZAM particles rely on the use of the endosomal and exosomal pathways that in *Drosophila *ovaries are normally employed for vitellogenin release and uptake. There is now abundant evidence in the literature to indicate that retroviral Gag proteins interact with a variety of proteins involved in these pathways. Analysis of the role played by the Gag product in ZAM transfer, and its potential interaction with cellular factors necessary for the vitellin traffic at this stage of oogenesis is under investigation.

## Methods

### Fly stocks

The U line is from the collection of the Institut National de la Santé et de la Recherche Médicale U384. The following female sterile mutations were used: *fs(2)A17*, *fs(1)1163*, *yl *are from the Bloomington stock center.

### Genetics crosses

All crosses were performed at 25°C. Flies were grown on standard media. The following crosses were performed. Males with the genotype X^u^; fs(2)A17/cyo; +/Tm3 were crossed with females X^u^/X^u^; fs(2)A17/Cyo; Tm3/Ap, and males X^u^; fs(2)A17/cyo; +/Tm3 were crossed with females X^u^/X^u^; fs(2)A17/cyo; +/Tm3. Ovaries of the female X^u^/X^u^; fs(2)A17/fs(2)A17 were dissected and examined by confocal or electron microscopy. Males fs(1)1163; +/+; +/+ or yl^-^; +/+; +/+ were crossed to females X^u^/X^u^; Cyo Tm3/Ap. The resulting F1 Females with the following genotype X^u^/fs(1)1163; +/Cyo; +/Tm3 or X^u^/yl^-^; +/Cyo; +/Tm3 were dissected and analyzed.

### Immunofluorescence

Ovaries were dissected in cold PBS and fixed in 5% formaldehyde-PBS for 20 min. After, two washes in PBS, ovaries were permeabilized 1 hour in PBS-Triton 0.5%. Primary antibodies pAbGagZAM and a rat anti-YL targeting *yolkless *receptor were then added at 1/100 and 1/200 respectively, and incubated overnight at 4°C. Secondary antibodies (goat anti-rabbit Cy3 and goat anti-rat alexa 488) were added at 1/100 and 1/200 respectively during 3 hours. After 3 washes in PBS-Triton 0.1%, slides were mounted in PBS/glycerol (1:1) and observed with a confocal fluorescent microscope (Olympus).

### Ultrastructural studies

For ultrastructural studies 2- to 3-day-old flies were dissected in PBS, and the ovaries were quickly fixed for 2 h in ice-cold 5% glutaraldehyde – 4% formaldehyde in 0.1 M cacodylate buffer at pH 7.2. Individual ovarian follicles were separated from the ovaries while in the fixative. Following a prolonged rinse in the same buffer, the ovarian follicles were postfixed for 2 h in 1% Osmium tetroxide in 0.1 M cacodylate buffer at pH 7.2 and rinsed again in the same buffer. Ovarian follicles were then dehydrated in a graded series of alcohols, passed through propylene oxide, and eventually polymerized in epoxy resin for 3 days at 60°C.

For immunocytochemical detection of viral antigens, ovarian follicles were fixed for 2 h in 1% glutaraldehyde – 4% formaldehyde in 0.1 M buffer at pH 7.2. After dehydration in alcohols, ovarian follicles were embedded in Unicryl resins and allowed to polymerize under a UV lamp at 4°C for 3 days. Sections were obtained with an LKB ultramicrotome and mounted over uncoated nickel grids. To detect viral antigens by gold immunocytochemistry, a number of ovarian follicles were dissected and fixed in paraformaldehyde 1.6% plus glutaraldehyde 2.5% and then incubated, post-embedding, for 3 hrs in primary rabbit (pAbGag) antibodies diluted 1:500 in PBS. Ovarian follicles were then thoroughly rinsed in PBS and incubated for an additional hour at room temperature with either gold-tagged secondary goat anti-rabbit immunoglobulin G (20 nM) diluted 1:200 in PBS. Grids were conventionally stained with uranyl acetate and lead citrate, and observed in a Jeol EM transmission electron microscope.

## Competing interests

The author(s) declare that they have no competing interests.

## Authors' contributions

EB performed the genetic crosses. EB and ART carried out the EM analysis. FA carried out the confocal analysis. BF, AMF and MM participated in the design of the study. FG and CV conceived of the study, and participated in its design and coordination and helped to draft the manuscript. All authors read and approved the final manuscript.
